# Equal Neutralization Potency of Antibodies Raised against Abrin Subunits

**DOI:** 10.3390/antib9010004

**Published:** 2020-02-06

**Authors:** Yoav Gal, Anita Sapoznikov, Reut Falach, Ohad Mazor, Ron Alcalay, Eytan Elhanany, Moshe Aftalion, Sharon Ehrlich, Chanoch Kronman, Tamar Sabo

**Affiliations:** 1 Department of Biochemistry and Molecular Genetics, Israel Institute for Biological Research, Ness-Ziona 76100, Israel; anitas@iibr.gov.il (A.S.); reutf@iibr.gov.il (R.F.); rona@iibr.gov.il (R.A.); eytanel@gmail.com (E.E.); moshea@iibr.gov.il (M.A.); sharone@iibr.gov.il (S.E.); chanochk@iibr.gov.il (C.K.); tamars@iibr.gov.il (T.S.); 2 Department of Infectious Diseases, Israel Institute for Biological Research, Ness-Ziona 76100, Israel; ohadm@iibr.gov.il

**Keywords:** abrin, ricin, chimeric toxins, A-subunit, B-subunit, polyclonal antibodies

## Abstract

Abrin and ricin are potent AB toxins, which are considered biological threats. To date, there are no approved treatments against abrin or ricin intoxications. Previously, we showed that the administration of polyclonal anti-abrin antibodies to mice that were intranasally exposed to abrin, even very late post-exposure, conferred an exceedingly high-level of protection, while following ricin intoxication, similar treatment with anti-ricin antibodies resulted in negligible survival rates. To probe this unexpected difference in protection ability, we first examined whether the efficient anti-abrin-induced protection was due to neutralization of the A-subunit responsible for the catalytic effect, or of the B-subunit, which enables binding/internalization, by evaluating the protection conferred by antibodies directed against one of the two subunits. To this end, we generated and immunized rabbits with chimeric toxins containing a single abrin subunit, A_abrin_B_ricin_ in which abrin A-subunit was linked to ricin B-subunit, and A_ricin_B_abrin_ in which ricin A-subunit is linked to abrin B-subunit. Here, we show that antibodies raised against either A_abrin_B_ricin_ or A_ricin_B_abrin_ conferred exceptionally high protection levels to mice following intranasal exposure to a a lethal dose of abrin, suggesting that the high level of protection conferred by anti-abrin antibodies is not related to the neutralization of a particular subunit.

## 1. Introduction

Abrin and ricin, plant toxins derived from rosary peas (*Abrus precatorius*) and castor bean plant (*Ricinus communis*), respectively, belong to the type 2 ribosome inactivating (RIP-2) group of proteins. The heterodimeric glycoprotein of both toxins comprises subunit A, carrying the enzymatic activity of the toxin, and subunit B, which is a lectin that binds to eukaryotic cell surfaces via galactose residues and mediates toxin internalization [1]. Upon entry to the cytosol, the A-subunits of both ricin and abrin inactivate ribosomes irreversibly by site-specific depurination of a specific adenine nucleotide within the 28S rRNA, thereby precipitating cessation of cellular protein synthesis [2]. Due to their high availability, ease of preparation and high toxicity, ricin and abrin are considered potential bioterror agents and are classified as Category B agents by the US Center for Disease Control and Prevention (CDC). To date, there are no approved post-exposure therapeutic countermeasures for either toxin. 

The toxicity of ricin and abrin depends on the route of exposure, inhalatory exposure being considered the most fatal [2]. The clinical manifestation following intranasal exposure of mice to these toxins is the onset of a localized yet severe pulmonary edematous inflammation, which is accompanied by massive recruitment of neutrophils to the lungs and onset of a turbulent pro-inflammatory cytokine storm within this organ. Pulmonary (intranasal) ricin and abrin intoxications in mice, are similar with regard to pathological features and kinetics [3,4]. However, despite their resemblance in morbidity and mortality, the ability to protect mice against ricin and abrin intoxications by post-exposure antibody-mediated treatment differs radically. In the case of lethal ricin intoxication, rabbit-derived polyclonal anti-ricin antibody-based treatment up to three hours post exposure conferred protection to ≥90% of the mice, nonetheless following treatment at 24 hours survival rates declined dramatically, to 34% [3]. When antibody treatment was administered at 48 hours post exposure to ricin, protection was no more than marginal [5]. In sharp contrast, the administration of polyclonal anti-abrin antibodies to mice intranasally exposed to a lethal dose of abrin, led to very high survival rates (~70%–80%), even when the antibodies were applied as late as 72 hours after intoxication [4]. 

The goal of the present work was to elucidate whether the efficient protection by polyclonal anti-abrin antibodies can be attributed to the neutralization of a single subunit, in other words, to define the critical neutralizing event that confers high-level of protection: prevention of toxin binding via the B subunit or abolishment of the A subunit-related catalytic activity. A specific conclusion could assist us, for example, to produce a subunit-directed abrin vaccine, which would be as efficient as holotoxin-based vaccine, but much safer. In addition, determining the subunit whose neutralization is critical for protection could assist in designing a more effective anti-ricin immunization protocol. 

Production of effective polyclonal antibodies against a specific subunit of the abrin (or ricin) toxin is complicated by the tendency of isolated subunits to adopt an unstable conformation (in particular, B_abrin_ is insoluble in aqueous solutions when separated from the A_abrin_, due to its hydrophobicity [6]). To overcome this difficulty, we incorporated each of the abrin subunits into a dimeric AB molecular fold by generating two chimeric heterologous toxins, A_abrin_B_ricin_ and A_ricin_B_abrin_, utilizing the monomeric subunit constituents of abrin and ricin. These chimera toxins served as antigens for rabbit vaccination, which resulted in elicitation of antibodies against each of the anti-abrin-subunits.

In the present study, we found that anti-A_abrin_ and anti-B_abrin_ antibodies were equally effective in recognizing and neutralizing abrin in-vitro. Highly efficient protection of mice against pulmonary abrin intoxication, comparable to that conferred by anti-abrin holotoxin antibodies, was obtained by post-exposure application of either one of the anti-subunit directed antisera. These findings strongly suggest that the neutralization of either A_abrin_ or B_abrin_ is sufficient for achieving enhanced protection.

## 2. Materials and Methods

### 2.1. Extraction and Purification of Abrin and Ricin

Crude abrin was prepared from *Abrus precatorius* seeds essentially as described previously [7,8]. Briefly, seed kernels were soaked in 5% acetic acid/phosphate buffer (Na_2_HPO_4_, pH-7.4) overnight and homogenized in a Waring blender. Proteins obtained from 80% ammonium sulfate precipitation were centrifuged and dialyzed extensively against PBS. 

Crude ricin was prepared from seeds of endemic *Ricinus communis*, essentially as described before [9]. Briefly, seeds were homogenized in a Waring blender in 5% acetic acid/phosphate buffer (Na_2_HPO_4_, pH-7.4), the homogenate was centrifuged and the clarified supernatant containing the toxin was subjected to ammonium sulfate precipitation (60% saturation). The precipitate was dissolved in PBS and dialyzed extensively against the same buffer. 

For the preparation of purified toxins, crude abrin and ricin were loaded consecutively on 2 columns, the first column containing activated Sepharose which binds and thereby depletes the *Abrus precatorius* Agglutinin (APA) and *ricinus communis* agglutinin (RCA), respectively, and the second column, containing α-lactose (lactamyl) agarose (Sigma-Aldrich, Rehovot, Israel), which binds the toxins. Proteins bound to the lactamyl agarose column were eluted with 0.5M Galactose in PBS. 

### 2.2. Subunit Purification 

Purified abrin was loaded on a lactamyl agarose column (1 mg pure abrin:2 mL lactamyl agarose) and the column was washed with 1M Trizma-HCl pH-8 (Sigma-Aldrich, Rehovot, Israel), which was diluted 1:10 in PBS (abrin washing buffer). For toxin reduction and collection of A_abrin_, the column was rinsed with 1% v/v β-mercaptoethanol in abrin washing buffer. Each 1 mL A_abrin_ was neutralized with 25 µL of 1M KH_2_PO_4_. The column was washed with PBS (pH 7), and B_abrin_ was eluted with 0.5M galactose in the presence of 1% v/v β-mercaptoethanol. A_abrin_ and B_abrin_ were concentrated in 10 kDa cutoff Amicon-Ultra centrifugal filters. 

Purified ricin was loaded on a lactamyl agarose column (1 mg pure ricin:1 mL lactamyl agarose) and the column was washed with 1M Trizma-HCl pH-9 (Sigma-Aldrich, Rehovot, Israel) diluted 1:10 in PBS (ricin washing buffer). For toxin reduction and the collection of A_ricin_, the column was rinsed with 1% v/v β-mercaptoethanol (Sigma-Aldrich, Rehovot, Israel) in ricin washing buffer. Each 1 mL A_ricin_ was neutralized with 100 µL of 1M KH_2_PO_4_. The column was then washed with PBS (pH-7), and B_ricin_ was eluted with 0.5M galactose (Sigma-Aldrich, Rehovot, Israel), in the presence of 1% v/v β-mercaptoethanol. A_ricin_ and B_ricin_ were concentrated in 10 kDa cutoff Amicon-Ultra centrifugal filters (Mercury, Rosh Haayin, Israel). 

All subunit purification processes were conducted at 4 °C, and the subunits were stored under this temperature until further used.

### 2.3. Preparation of Chimeric Toxins

Chimera A_abrin_B_ricin_ preparation: the subunits were mixed (20% excess A_abrin_) and extensively dialyzed (10 kDa cutoff) against 10mM phosphate buffer (1M phosphate buffer solution, pH-7.4, diluted 1:100 in DDW) containing 100 mM galactose, at 4 °C for 7 days, and additional 2 days against the same buffer without galactose. A_abrin_B_ricin_ was loaded on a lactamyl agarose column and the column was washed with PBS in order to remove residual monomeric A_abrin_. A_abrin_B_ricin_ eluted with 0.5M galactose in PBS was dialyzed against PBS and kept frozen until used.

Chimera A_ricin_B_abrin_ preparation: the subunits were mixed (20% excess Aricin) and extensively dialyzed (10 kDa cutoff) against PBS for 2 days at 4 °C. A_ricin_B_abrin_ was loaded on a lactamyl agarose column and the column was washed with PBS in order to remove residual monomeric A_ricin_. A_ricin_B_abrin_ eluted with 0.5M galactose in PBS was dialyzed against PBS and kept frozen until used.

### 2.4. Gel Electrophoresis

Samples were visualized using Coomassie Blue stained non-reducing 10% polyacrylamide gel, which was subjected to sodium dodecylsulphatepolyacrylamide gel electrophoresis (SDS-PAGE) under reducing or non-reducing conditions.

### 2.5. ELISA Titer Determination

Maxisorp 96-well plates (Nunc, Sigma-Aldrich, St. Louis, MO, USA) were coated overnight with 2 µg/mL of abrin in 50 mM pH-9.6 Carbonate-Bicarbonate Buffer (Sigma, C3041), then washed and blocked with PBST buffer (0.05% Tween 20, 2% BSA in PBS) for one hour. Antisera were then added to the plates for a one-hour incubation. The plates were then washed with PBST and incubated with the detecting antibody, AP conjugated goat anti-rabbit (Jackson, 111-055-003) followed by detection with SIGMAFAST p-Nitrophenyl phosphate (Sigma, N1891).

### 2.6. In-Vitro Abrin Neutralization Assay

Ub-FL cells (HeLa cells stably expressing ubiquitin-luciferase [10]), a kind gift from Professor Piwnica-Worms (University of Texas, MD Anderson Center, Austin, TX, USA), were seeded in a 96-well plate (1.5 × 10^4^ cells/well) and incubated over night at 37 °C. Cell-culture medium was removed and serial dilutions of anti-holotoxin_abrin_, anti-A_abrin_, or anti-B_abrin_ antisera in the presence or absence of abrin (7 ng/mL) were added to the plate. Twenty four hours later, cell-culture medium was removed, and the cell medium containing the proteasome inhibitor MG132 (Sigma, C2211 1 µM) was added for another hour. After medium aspiration, cells were lysed by the addition of 50 µL lysis buffer (Promega, E1941). Residual luciferase activity was determined by mixing equal volumes of the cell lysate and luciferase assay reagent (Promega, E1483) followed by immediate measurement of the luminescence levels. Residual luciferase activity was expressed as percent of luminescence levels obtained from untreated cells.

### 2.7. Epitope Recognition

Maxisorp 96-well microtiter plates were coated overnight with 5µg/ mL Streptavidin (Sigma, S0677), then washed and blocked as described above. Biotin labeled 15aa length peptides (Table S1) (5 µg/mL in PBST) were then added for 20 minutes. The plates were washed, anti-holotoxin_abrin_/anti-A_abrin_B_ricin_/anti-A_ricin_B_abrin_ antisera were diluted in PBST and added to the plates for one-hour incubation. The plates were washed and incubated with AP conjugated anti-rabbit antibodies (Jackson, 111-055-003) for 15 minutes followed by detection with SIGMAFAST p-Nitrophenyl phosphate (Sigma, N1891).

### 2.8. Animal Studies

Animal experiments were performed in accordance with the Israeli law and were approved by the Ethics Committee for animal experiments at the Israel Institute for Biological Research. The treatment of animals was in accordance with regulations outlined in the USDA Animal Welfare Act and the conditions specified in the Guide for Care and Use of Laboratory Animals (National Institute of Health, 1996). New Zealand white rabbits (Charles River Laboratories Ltd., UK) weighing 2.5 to 3 kg were immunized in order to produce rabbit-derived polyclonal antibodies. Female CD-1 mice (Charles River Laboratories Ltd., UK) weighing 27–32 g were used for survival studies. 

Prior to all studies in mice and rabbits, the animals were habituated to the experimental animal unit for 5 days. All mice were housed in filter-top cages in an environmentally controlled room and maintained at 21 ± 2 °C and 55 ± 10% humidity. Lighting was set to mimic a 12/12 hours dawn to dusk cycle. Animals had access to food and water *ad libitum*.

### 2.9. Polyclonal Anti-Chimera Antibody Production

Rabbits were immunized with A_abrin_B_ricin_ or A_ricin_B_abrin_ in a stepwise manner, injection 1 containing 1 µg toxin in Freund’s adjuvant, and injections 2–5 (with incomplete Freund’s adjuvant) in increasing concentrations (4, 25, 50, and 100 µg toxin/rabbit) with 4-week intervals between injections. Blood samples were collected (1 week after injection) to ascertain anti-chimera antibody titer build-up. 

### 2.10. In-Vivo Protection Assay

Mice were anesthetized by an intraperitoneal (i.p.) injection of ketamine (1.9 mg/mouse, Vetoquinol, Lure, France) and xylazine (0.19 mg/mouse, Eurovet Animal Health, AD Bladel, The Netherlands), and intoxicated by intranasal instillation (2 × 25 µL) of 2LD_50_ of abrin (8 µg/kg). For antibody treatment 100 µL of the relevant rabbit-derived polyclonal antibodies were applied intravenously at 48 hours following abrin intoxication. Mortality was monitored over 14 days and Kaplan-Meier analysis was performed for survival curves. 

## 3. Results

### 3.1. Characterization of the Chimeric Toxins

**Figure 1:**
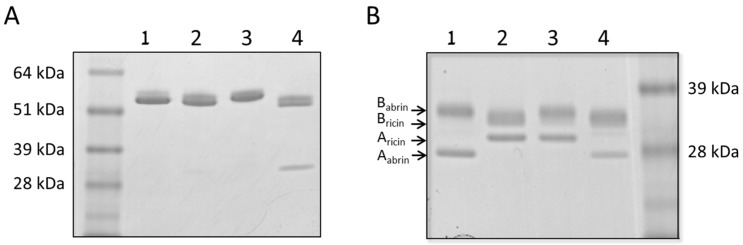
Gel electrophoresis of chimera abrin/ricin toxins. SDS-PAGE of: Reconstituted abrin (lane 1), Reconstituted ricin (lane 2), Chimera toxin A_ricin_B_abrin_ (lane 3), and Chimera toxin A_abrin_B_ricin_ (lane 4) was performed (**A**) in the absence of reducing agent, or (**B**) in the presence of β-mercaptoethanol.

Chimeric abrin/ricin toxins were generated from isolated A and B-subunits of abrin and ricin, based upon a method developed by Olsnes et al. [11], with slight modifications (for details see “Materials and Methods”). Under non-reducing conditions, the chimeric A_abrin_B_ricin_ and A_ricin_B_abrin_ toxins, as well as reconstituted ricin and abrin toxin molecules generated by co-incubation of A and B-subunits of the same source, appear as a main band of approximately 60 kDa on SDS-PAGE (Figure 1A). The formation of A_abrin_B_ricin_ was incomplete, as indicated by detection of a weak ~30 kDa band, in addition to the major band. Interestingly, this observation was also reported previously [11]. Since excess A_-_subunits are removed prior to the final stage of the experimental protocol for chimeric toxin generation, this ~30 kDa band probably represents monomeric B_ricin_. As seen in Figure 1B, reduction with β-mercaptoethanol of reconstituted ricin and abrin (lanes 1 and 2, respectively) resulted in the generation of two typical major bands, somewhat different in molecular weights (the molecular weight of A_abrin_ is less than that of A_ricin_, the molecular weight of B_ricin_ is slightly less than that of B_abrin_). Reduction of the chimera toxins (lanes 3 and 4) resulted in electrophoretic migrating bands, which confirm the composition of each toxin.

### 3.2. Abrin Subunit Recognition and Neutralization 

To produce polyclonal antibodies directed specifically against A_abrin_ or B_abrin_, rabbits were immunized with the chimeric toxins, A_abrin_B_ricin_ and A_ricin_B_abrin_, respectively. The immunization protocol included repeated injection with increasing antigen doses, at four-week intervals, up to 100 µg antigen/rabbit. Hyperimmune sera directed against each of the chimeric toxins was collected and characterized for its ability to recognize A_abrin_ or B_abrin_ in an enzyme linked immunosorbent assay (ELISA). Accordingly, increasing concentrations of either the anti-chimera antisera were incubated on abrin-coated plates, after which abrin-bound antibodies were detected using secondary anti-rabbit antibodies. The two antisera displayed ELISA titers of similar magnitude against the corresponding abrin subunits, 4.5 × 10^5^ and 8 × 10^5^ ELISA units, against A_abrin_ and B_abrin_, respectively (Table 1). Next, we asked whether these anti-subunit antibodies possess the capability to neutralize abrin in an in-vitro cell culture system that may potentially predict their efficacy in-vivo. In this assay, the neutralization potency of an antiserum is measured by monitoring its ability to block abrin-induced luciferase synthesis arrest in HeLa Ub-FL cells [12,13]. Abrin was incubated with increasing concentrations of the relevant antiserum, and added to the Ub-FL cells. Twenty four hours later, residual intracellular luciferase levels were measured and neutralizing antibody titers were determined for each antisera. As can be seen (Table 1), both subunit-directed anti-chimera antibodies possess the ability to effectively neutralize abrin in cell culture (~1 × 10^3^ neutralizing antibody titers for both anti-chimera antitoxins).

### 3.3. Abrin Linear-Epitope Recognition by Anti-A_abrin_- and Anti-B_abrin_- Polyclonal Antibodies

**Figure 2:**
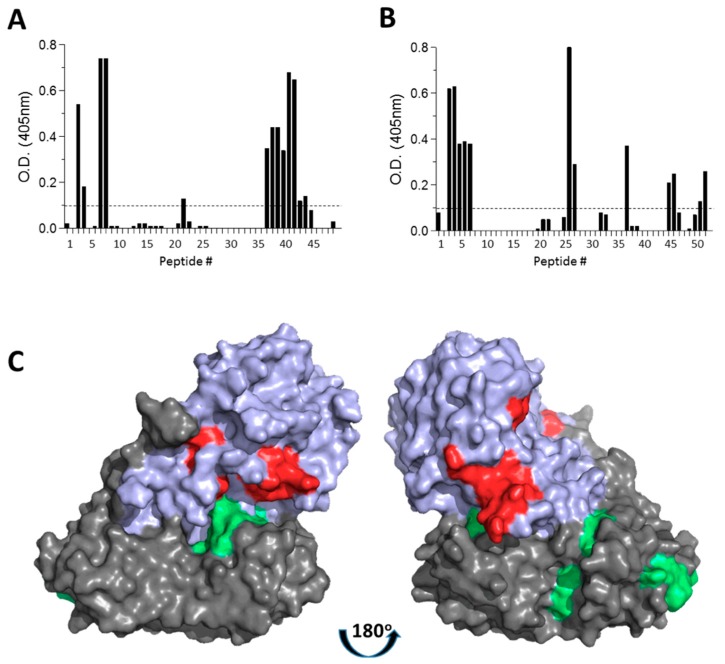
Linear epitopes recognized by anti-abrin-subunit antisera. Linear 15-mer peptides spanning (**A**) A_abrin_ or (**B**) B_abrin_ were reacted with either anti-A_abrin_B_ricin_ antisera or anti-A_ricin_B_abrin_ antisera, respectively. Dash line represents background binding. (**C**) Schematic representation of the linear epitopic regions recognized by anti-A_abrin_ antibodies (red) and anti-B_abrin_ antibodies (green) are depicted on the crystal structure of abrin (PDB 1abr) using PyMol. A_abrin_ and B_abrin_ are represented by cyan and gray colors, respectively.

We have recently determined the immunodominant epitopes of abrin in sera of rabbits that were immunized with abrin (manuscript in preparation). We now sought to characterize the antibody response elicited against A_abrin_ and B_abrin_, when these abrin-derived subunits are embedded within the chimeric toxins. By allowing each of the anti-chimera antisera to react with overlapping 15-mer peptides spanning the protein sequence of either A_abrin_ or B_abrin_, we could determine to which linear epitopes each sera is directed (Figure 2). It was found that the anti-A_abrin_B_ricin_ antiserum recognizes five epitopes of A_abrin_ (Table 2). While epitopes 1, 2, and 4 were previously mapped as immunodominant epitopes of anti-abrin antisera, epitopes 3 and 5 were discerned only by anti-A_abrin_B_ricin_ antisera. It should be noted however, that the signals of the sera reacting with the three known epitopes were high whereas the signal with the hitherto undetected two epitopes was relatively low (close to background level), suggesting that these two epitopes have minor contribution to the overall activity of the sera toward abrin. Mapping the epitopes of the anti-A_ricin_B_abrin_ reveals six epitopes (Table 2) all of which were previously mapped as immunodominant epitopes of B_abrin_. It should be noted that epitope 9 is based on signal of the sera binding to a single peptide (No. 37). The sequence DGSI also appears as a part of a larger epitope sequence (epitope 10), suggesting that the binding of the antibodies to peptide 37 is due to possible cross-reactivity with this sequence. Taken together, these results indicate that the overall structure and immunogenicity of the chimeric subunits are similar to that of the native toxin. 

### 3.4. Post-Exposure Treatment of Abrin Intoxicated Mice with Anti-Abrin Subunit-Directed Antisera

As the goal of this study was to elucidate which abrin subunit needs to be neutralized in-vivo to promote the observed high level of protection following pulmonary abrin-intoxication [4], mice were intranasally exposed to 2LD_50_ of abrin (8 µg/kg), and anti-abrin or anti-abrin-subunit (anti-chimera) antibodies (100 μL intravenously) were administered at 48 hours post exposure. Control intoxicated mice were not subjected to antibody treatment. The data in Table 3 establish that, as in the case of anti-abrin holotoxin antibody treatment, late administrations of either anti-A_abrin_ or anti-B_abrin_ antisera conferred very high protection levels (≥90%) against a lethal abrin intoxication. 

## 4. Discussion

Previous studies carried out in our laboratory, demonstrated that application of anti-abrin antibodies conferred extremely high survival rates to intranasally abrin-intoxicated mice, even when treatment was performed at very late time after exposure to a lethal dose of the toxin [4]. In contrast, late anti-ricin antibody treatment following lethal ricin intoxication resulted in insignificant protection [3]. This unexpected sharp dissimilarity in antibody-induced protection could stem from a difference in the quality of the anti-abrin and anti-ricin antibody preparations, or from differences in disease progression following exposure to the two toxins. 

We have previously shown that anti-ricin and anti-abrin ELISA- and neutralizing- antibody titers, as well as the apparent affinities of the specific fraction of either anti-ricin or anti-abrin antibodies, were very similar [4]. However, this data could not rule out the possibility that neutralization of an essential epitope within a specific subunit, or perhaps neutralization of the functional activity of one of the two subunits, toxin catalytic activity or lectin binding of subunit A and B, respectively, is obligatory for obtaining high-level protection. To evaluate such subunit-related neutralization effects, we sought to produce anti-abrin polyclonal antibodies directed specifically against A_abrin_ or B_abrin_. An obvious option for generating such antibodies, would be to immunize rabbits with isolated monomeric A_abrin_ or B_abrin_, however isolated individual subunits, originating from an AB dimeric context, are unstable and their conformational structure is drastically impaired [14]. Indeed, export of the abrin (or ricin) monomeric A-subunit from the endoplasmic reticulum (ER) into the cytosol relies on the fact that following toxin reduction in the ER, the isolated A-chain is identified as a misfolded protein, thereby allowing its retrieval to the cytosol via the ER degradation pathway [15,16]. In this regard, it has been suggested that ribosome depurination within the cytosol by monomeric A-subunit, requires its refolding, with the aid of the Hsc70 chaperone, to a catalytic conformation [17]. With respect to the B-subunit, it has been documented that B_abrin_ is insoluble in aqueous solutions when separated from the A_abrin_, due to its hydrophobicity [6]. Accordingly, isolated subunit vaccination may lead to the production of antibodies that could not recognize and neutralize the holotoxin, resulting in inability to protect against in-vivo intoxications. Indeed, when recombinant A_abrin_ was used as the immunizing antigen for production of anti-A_abrin_ mAbs, in-vivo neutralization of abrin could be demonstrated only when these mAbs were applied prophylactically, while no post-exposure effects were reported [18,19]. MAbs directed against A_abrin_-*abrus precatorius agglutinin* (APA) chimeric toxin (1–123 amino acids of A_abrin_ linked to 124–175 amino acids of APA), in which A_abrin_ is not in an AB holotoxin context, were also found to be protective prophylactically, without any reports regarding post exposure effects [20]. Consequently, to produce an abrin-subunit specific antigen possessing proper abrin-subunit conformational structure for rabbit immunization, we generated chimeric abrin-ricin toxins composed of A_abrin_ or B_abrin_ linked via disulfide bond to their non-homologous counterparts, subunit B_ricin_ or A_ricin_, respectively. Characterization of the two antisera raised against the two chimeric abrin/ricin toxins demonstrated that these recognized their cognate subunits to a similar extent and that both anti-chimeric toxin antibodies possessed potent in-vitro anti-cytotoxicity abilities. Most importantly, we found that following intranasal exposure of mice to abrin, both subunit-directed antisera, anti-A_abrin_B_ricin_ (anti-A_abrin_) and anti-A_ricin_B_abrin_ (anti-B_abrin_), were highly protective (≥90% survival) even when treatments were applied as late as 48 hours following intoxication. This high level of protection is due to the antibodies elicited against the abrin subunit of the chimeric toxins and not to those formed against the ricin subunit, since anti-ricin antibodies were completely ineffective when given to mice even at early hours following intranasal exposure to a lethal dose (2LD_50_) of abrin (data not shown). 

Taken together, it seems unlikely that the difference in protection conferred by anti-abrin and anti-ricin antibodies against abrin and ricin intoxications, respectively, is related to variances in the quality of the two antibody preparations. Rather, it seems that the striking difference in the ability to protect against ricin- and abrin- challenges stems from dissimilarities in disease progression. Supporting this argument, we have previously shown that following intranasal intoxication, the in-vivo catalytic performance of abrin, i.e., ribosomal depurination of pulmonary tissue, is significantly lower than that observed following ricin intoxication. In particular, the depurination level of pulmonary epithelial cells was markedly reduced following abrin intoxication, approximately five times lower than after ricin intoxication (16% and 81% depurination following exposure to abrin and ricin, respectively [21]). We further determined by FACS analysis that the epithelial cell damage following intoxications with abrin (unpublished data) and ricin [22] was mostly confined to the type II epithelial cells, responsible for lung tissue regeneration [23]. The injury to the epithelial cell sub-population was correlative to the trend described above for the whole-epithelial population, namely, the specific insult to the type II cells following abrin intoxication was less prominent than the damage observed following ricin exposure (unpublished data). Thus, anti-abrin and anti-ricin antibodies may halt further toxin-induced damage in an equally efficient manner, yet the anti-abrin antibodies will provide greater protection to abrin-intoxicated subjects due to the reduced levels of irreversible cellular damage inflicted by this particular toxin at a given post-exposure time-point. Furthermore, the notion that antibody-related protection ability is mostly due to toxin pathology progression and not to antibody quality, is in line with experiments recently carried out in our laboratory, in which mice were intoxicated with lethal doses of the chimeric toxins, and then treated with their corresponding anti-chimeric antibodies. Preliminary results suggest that significantly higher survival rates are achieved following late antibody treatment of mice that were intranasally intoxicated with the chimera toxin A_ricin_B_abrin_, even though both anti-A_abrin_B_ricin_ and anti-A_ricin_B_abrin_ conferred equally high levels of protection to abrin-intoxicated mice. Future studies will be required for characterization of the different damage patterns in the pulmonary cell subpopulations following intranasal intoxications with the two chimeric toxins.

In parallel to the results described above, recent studies carried out in our laboratory established that the low-density lipoprotein Receptor Related-protein 1 (LRP-1) is the major receptor for ricin in murine lung cells (Falach et al, manuscript in preparation). A different receptor for abrin (and A_ricin_B_abrin_), if indeed detected, may further explain the dissimilarity between the disease progressions following pulmonary abrin and ricin intoxications.

In summary, both anti-A_abrin_- and anti-B_abrin_ directed rabbit-derived polyclonal antibodies confer extremely effective protection against abrin lethality when administered lately following intranasal abrin intoxication in mice, allowing us to conclude that the significant difference in protection levels following antibody treatment after intranasal abrin and ricin intoxications stems from a dissimilarity in disease progression inflicted by these two toxins. 

## Figures and Tables

**Table 1:** Anti-abrin titers of rabbit-derived anti-A_abrin_B_ricin_ and anti-A_ricin_B_abrin_ antisera.

Antiserum (Relevant Abrin Subunit)	ELISA-Antibody Titers (× 10^5^)	Neutralizing-Antibody Titers (× 10^3^)
**Anti-AabrinBricin (A)**	4.5 ± 0.1	1.1 ± 0.1
**Anti-AricinBabrin (B)**	8.1 ± 0.2*	0.9 ± 0.3

* *p* < 0.05 when compared to Anti-AabrinBricin ELISA-antibody titer.

**Table 2:** anti-A_abrin_B_ricin_ and anti-A_ricin_B_abrin_ antisera immunodominant epitopes of abrin.

Epitope No.	Subunit	Epitope Sequence	Epitope Location
1	Aabrin	KQFIEALR	18–25
2		IPVLPDP	36–42
3		YGTYGDL	110–116
4		QPDAAMISLE	186–195
5		LTIRN	219–223
6	Babrin	VRIGGRDG	16–23
7		NGYHNG	31–36
8		QGWRTGN	134–140
9		DGSI	183–186
10		WVKFNDGSI	221–229
11		QIWLTLF	261–267

**Table 3:** Survival rates of mice intoxicated with abrin and subjected to antibody treatment.

	Survival	
Antibody treatment	%	Survivors/Total
**Control**	0	0/10
**Anti-Abrin**	90	9/10
**Anti-AabrinBricin**	90	9/10
**Anti-AricinBabrin**	95	19/20

## Supplementary Materials

The following are available online at https://www.mdpi.com/2073-4468/9/1/4/s1, Table S1: List of 15-mer peptides of Aabrin and Babrin.

## References

[1] Olsnes S. (2004). The history of ricin, abrin and related toxins. Toxicon.

[2] Audi J., Belson M., Patel M., Schier J., Osterloh J. (2005). Ricin poisoning: a comprehensive review. JAMA.

[3] Gal Y., Mazor O., Alcalay R., Seliger N., Aftalion M., Sapoznikov A., Falach R., Kronman C., Sabo T. (2014). Antibody/doxycycline combined therapy for pulmonary ricinosis: attenuation of inflammation improved survival of ricin-intoxicated mice. Toxicol. Rep..

[4] Sabo T., Kronman C., Mazor O. (2016). Ricin-Holotoxin-Based Vaccines: Induction of Potent Ricin-Neutralizing Antibodies. Methods Mol. Biol..

[5] Gal Y., Sapoznikov A., Falach R., Ehrlich S., Aftalion M., Kronman C., Sabo T. (2017). Total Body Irradiation Mitigates Inflammation and Extends the Therapeutic Time Window for Anti-Ricin Antibody Treatment against Pulmonary Ricinosis in Mice. Toxins.

[6] Krupakar J., Swaminathan C.P., Das P.K., Surolia A., Podder S.K. (1999). Calorimetric studies on the stability of the ribosome-inactivating protein abrin II: effects of pH and ligand binding. Biochem. J..

[7] Kumar M.S., Karande A.A. (2016). A monoclonal antibody to an abrin chimera recognizing a unique epitope on abrin A chain confers protection from abrin-induced lethality. Hum. Vaccin. Immunother.

[8] Falach R., Sapoznikov A., Gal Y., Israeli O., Leitner M., Seliger N., Ehrlich S., Kronman C., Sabo T. (2016). Quantitative profiling of the in-vivo enzymatic activity of ricin reveals disparate depurination of different pulmonary cell types. Toxicol. Lett..

[9] Sapoznikov A., Falach R., Mazor O., Alcalay R., Gal Y., Seliger N., Sabo T., Kronman C. (2015). Diverse profiles of ricin-cell interactions in the lung following intranasal exposure to ricin. Toxins.

[10] Mason R.J. (2006). Biology of alveolar type II cells. Respirology.

[11] Hegde R., Podder S.K. (1992). Studies on the variants of the protein toxins ricin and abrin. Eur. J. Biochem..

[12] Olsnes S., Pihl A. (1973). Isolation and properties of abrin: a toxic protein inhibiting protein synthesis. Evidence for different biological functions of its two constituent-peptide chains. Eur. J. Biochem..

[13] Lin J.Y., Liu S.Y. (1986). Studies on the antitumor lectins isolated from the seeds of Ricinus communis (castor bean). Toxicon.

[14] Luker G.D., Pica C.M., Song J., Luker K.E., Piwnica-Worms D. (2003). Imaging 26S proteasome activity and inhibition in living mice. Nat. Med..

[15] Olsnes S., Pappenheimer A.M., Meren R. (1974). Lectins from Abrus precatorius and Ricinus communis. II. Hybrid toxins and their interaction with chain-specific antibodies. J. Immunol..

[16] Gal Y., Alcalay R., Sabo T., Noy-Porat T., Epstein E., Kronman C., Mazor O. (2015). Rapid assessment of antibody-induced ricin neutralization by employing a novel functional cell-based assay. J. Immunol. Methods.

[17] Mechaly A., Alcalay R., Noy-Porat T., Epstein E., Gal Y., Mazor O. (2018). Novel Phage Display-Derived Anti-Abrin Antibodies Confer Post-Exposure Protection against Abrin Intoxication. Toxins.

[18] Teter K. (2013). Toxin instability and its role in toxin translocation from the endoplasmic reticulum to the cytosol. Biomolecules.

[19] Lord J.M., Roberts L.M., Lencer W.I. (2005). Entry of protein toxins into mammalian cells by crossing the endoplasmic reticulum membrane: co-opting basic mechanisms of endoplasmic reticulum-associated degradation. Curr. Top. Microbiol. Immunol..

[20] Wesche J., Rapak A., Olsnes S. (1999). Dependence of ricin toxicity on translocation of the toxin A-chain from the endoplasmic reticulum to the cytosol. J. Biol. Chem..

[21] Spooner R.A., Hart P.J., Cook J.P., Pietroni P., Rogon C., Hohfeld J., Roberts L.M., Lord J.M. (2008). Cytosolic chaperones influence the fate of a toxin dislocated from the endoplasmic reticulum. Proc. Natl. Acad. Sci. USA.

[22] Bagaria S., Ponnalagu D., Bisht S., Karande A.A. (2013). Mechanistic insights into the neutralization of cytotoxic abrin by the monoclonal antibody D6F10. PLoS ONE.

[23] Surendranath K., Karande A.A. (2008). A neutralizing antibody to the a chain of abrin inhibits abrin toxicity both *in-vitro* and *in-vivo*. Clin. Vaccine Immunol..

