# Supplementary material for: Equal Neutralization Potency of Antibodies Raised against Abrin Subunits
Source: Antibodies (Basel). 2020 Feb 6;9(1):4. doi: 10.3390/antib9010004 (PMC7148520; doi:10.3390/antib9010004)
Supplement: Supplementary file 1 [file antibodies-09-00004-s001.pdf]

Table S1: List of 15-mer peptides of Aabrin and Babrin

	ATA		ATB
1	EDRPIKFSTEGATSQ	1	IVEKSKICSSRYEPT
2	KFSTEGATSQSYKQF	2	KICSSRYEPTVRIGG
3	GATSQSYKQFIEALR	3	RYEPTVRIGGRDGMC
4	SYKQFIEALRERLRG	4	VRIGGRDGMCVDVYD
5	IEALRERLRGGLIHD	5	RDGMCVDVYDNGYHN
6	ERLRGGLIHDIPVLP	6	VDVYDNGYHNGNRII
7	GLIHDIPVLPDPTTL	7	NGYHNGNRIIMWKCK
8	IPVLPDPTTLQERNR	8	GNRIIMWKCKDRLEE
9	DPTTLQERNRYITVE	9	MWKCKDRLEENQLWT
10	QERNRYITVELSNSD	10	DRLEENQLWTLKSDK
11	YITVELSNSDTESIE	11	NQLWTLKSDKTIRSN
12	LSNSDTESIEVGIDV	12	LKSDKTIRSNGKCLT
13	TESIEVGIDVTNAYV	13	TIRSNGKCLTTYGYA
14	VGIDVTNAYVVAYRA	14	GKCLTTYGYAPGSYV
15	TNAYVVAYRAGTQSY	15	TYGYAPGSYVMIYDC
16	VAYRAGTQSYFLRDA	16	PGSYVMIYDCTSAVA
17	GTQSYFLRDAPSSAS	17	MIYDCTSAVAEATYW
18	FLRDAPSSASDYLFT	18	TSAVAEATYWEIWDN
19	PSSASDYLFTGTDQH	19	EATYWEIWDNGTIIN
20	DYLFTGTDQHSLPFY	20	EIWDNGTIINPKSAL
21	GTDQHSLPFYGTYGD	21	GTIINPKSALVLSAE
22	SLPFYGTYGDLERWA	22	PKSALVLSAESSSMG
23	GTYGDLERWAHQSRQ	23	VLSAESSSMGGTLTV
24	LERWAHQSRQQIPLG	24	SSSMGGTLTVQTNEY
25	HQSRQQIPLGLQALT	25	GTLTVQTNEYLMRQG
26	QIPLGLQALTHGISF	26	QTNEYLMRQGWRTGN
27	LQALTHGISFFRSGG	27	LMRQGWRTGNNTSPF
28	HGISFFRSGGNDNEE	28	WRTGNNTSPFVTSIS
29	FRSGGNDNEEKARTL	29	NTSPFVTSISGYSDL
30	NDNEEKARTLIVIIQ	30	VTSISGYSDLCMQAQ
31	KARTLIVIIQMVAEA	31	GYSDLCMQAQGSNVW
32	IVIIQMVAEAARFRY	32	CMQAQGSNVWMADCD
33	MVAEAARFRYISNRV	33	GSNVWMADCDSNKKE
34	ARFRYISNRVRVSIQ	34	MADCDSNKKEQQWAL
35	ISNRVRVSIQTGTAF	35	SNKKEQQWALYTDGS
36	RVSIQTGTAFQPDAA	36	QQWALYTDGSIRSVQ
37	TGTAFQPDAAMISLE	37	YTDGSIRSVQNTNNC
38	QPDAAMISLENNWDN	38	IRSVQNTNNCLTSKD
39	MISLENNWDNLSRGV	39	NTNNCLTSKDHKQGS
40	NNWDNLSRGVQESVQ	40	LTSKDHKQGSTILLM
41	LSRGVQESVQDTFPN	41	HKQGSTILLMGCSNG
42	QESVQDTFPNQVTLT	42	TILLMGCSNGWASQR
43	DTFPNQVTLTNIRNE	43	GCSNGWASQRWVFKN
44	QVTLTNIRNEPVIVD	44	WASQRWVFKNDGSIY
45	NIRNEPVIVDSLSHP	45	WVFKNDGSIYSLYDD
46	PVIVDSLSHPTVAVL	46	DGSIYSLYDDMVMDV
47	SLSHPTVAVLALMLF	47	SLYDDMVMDVKGSDP
48	TVAVLALMLFVCNPP	48	MVMDVKGSDPSLKQI
49	VAVLALMLFVCNPPN	49	KGSDPSLKQIILWPY
		50	SLKQIILWPYTGKPN
		51	ILWPYTGKPNQIWLT
		52	WPYTGKPNQIWLTLF
